# Troubleshooting for perforation due to proximal metal stent displacement in endoscopic ultrasound-guided hepaticogastrostomy

**DOI:** 10.1055/a-2590-1859

**Published:** 2025-05-26

**Authors:** Toji Murabayashi, Shinya Sugimoto

**Affiliations:** 1 37071 Department of Gastroenterology, Ise Red Cross Hospital, Ise, Japan

Partially covered self-expandable metal stents (PCSEMS) are frequently used in endoscopic ultrasound-guided hepaticogastrostomy (EUS-HGS). When the proximal end of the uncovered portion of a PCSEMS is positioned more proximally than the hepatic parenchyma, persistent bile leakage into the peritoneal cavity may occur even if the stent tip remains within the bile duct. Here, we report a technique to salvage this type of perforation. A 57-year-old man with unresectable hilar cholangiocarcinoma, who had previously undergone multiple transpapillary plastic stent placements in the bile duct, underwent EUS-HGS for recurrent biliary obstruction with gastric outlet obstruction ( Fig. 1 , Video 1 ). After puncturing with a 19-gauge needle and performing cholangiography, a PCSEMS with a 2 cm uncovered portion at the tip (Spring Stopper, 8 × 100 mm; TaeWoong Medical) was placed from B3 to the stomach. However, the stent migrated proximally during deployment, and the proximal end of the uncovered portion was inadvertently positioned within the peritoneal cavity. To address this complication, a guidewire was inserted into the PCSEMS and the bile duct via the side of the PCSEMS near the gastric wall by puncturing its membrane with a standard catheter under direct endoscopic visualization using a duodenoscope. Subsequently, a fully covered self-expandable metal stent (FCSEMS; 10 × 80 mm) was placed from the bile duct to the stomach, covering all uncovered portions of the PCSEMS. This successfully sealed the perforation and restored effective drainage ( Fig. 2 ). Matsubara et al. 1 previously reported managing the same complication by placing an additional FCSEMS using a different technique. They created an access route into the PCSEMS and bile ducts by puncturing with a 19-gauge needle through the gastric wall and hepatic parenchyma. Our approach offers advantages over their method, as it is not only technically simpler but also reduces the risk of gastric content leakage into the peritoneal cavity.

**Fig. 1:**
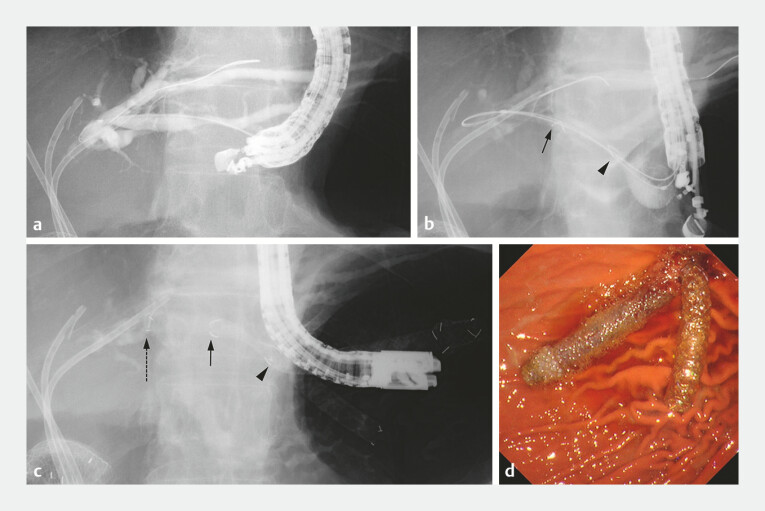
Fluoroscopic and endoscopic images during endoscopic ultrasound-guided hepaticogastrostomy. **a** Fluoroscopic image depicting the left intrahepatic bile duct filled with contrast medium. **b** Fluoroscopic image showing a PCSEMS. The distal edge of the PCSEMS and the boundary between the uncovered and covered portions are indicated by an arrow and an arrowhead respectively. **c** Fluoroscopic image showing an additional FCSEMS (HANAROSTENT Benefit) and PCSEMS. The distal edge of the PCSEMS, the boundary between the uncovered and covered portions, and the distal edge of the FCSEMS are indicated by an arrow, an arrowhead, and a dotted arrow, respectively. **d** Endoscopic image showing both the PCSEMS and FCSEMS within the stomach. Abbreviations: FCSEMS, fully covered self-expandable metal stent; PCSEMS, partially covered self-expandable metal stent.

Troubleshooting for perforation due to proximal metal stent displacement during endoscopic ultrasound-guided hepaticogastrostomy.Video 1

**Fig. 2:**
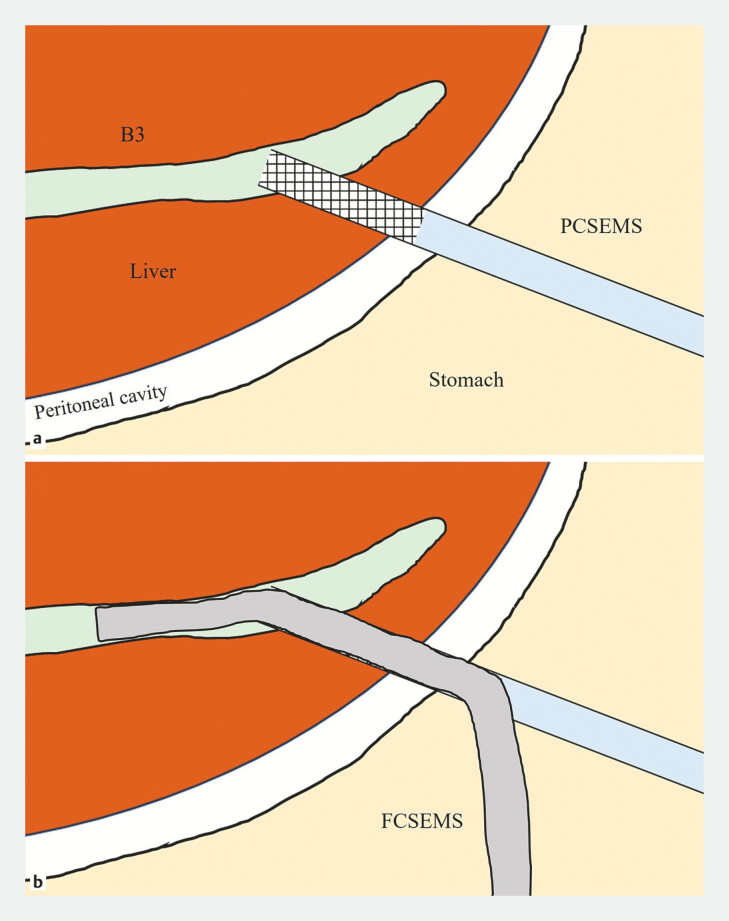
Schematic representation of the present case. **a** Persistent bile leakage into the peritoneal cavity through the uncovered portion of the PCSEMS. **b** The placement of an additional FCSEMS covering all uncovered portions of the PCSEMS, successfully sealing the perforation and restoring effective drainage. Abbreviations: FCSEMS, fully covered self-expandable metal stent; PCSEMS, partially covered self-expandable metal stent.

Endoscopy_UCTN_Code_CPL_1AL_2AD

## Notes

Endoscopy E-Videos https://eref.thieme.de/e-videos
*E-Videos* is an open access online section of the journal *Endoscopy* , reporting on interesting cases and new techniques in gastroenterological endoscopy. All papers include a high-quality video and are published with a Creative Commons CC-BY license. Endoscopy E-Videos qualify for HINARI discounts and waivers and eligibility is automatically checked during the submission process. We grant 100% waivers to articles whose corresponding authors are based in Group A countries and 50% waivers to those who are based in Group B countries as classified by Research4Life (see: https://www.research4life.org/access/eligibility/ ). This section has its own submission website at https://mc.manuscriptcentral.com/e-videos .
